# Redefining the Post-Mortem Investigation of Sudden Cardiac Death: Systematic Cardiac MR with Macroscopic and Histological Correlation from the Friuli Venezia Giulia Regional Registry

**DOI:** 10.3390/diagnostics16132067

**Published:** 2026-07-01

**Authors:** Lorenzo Pagnan, Alessandro Sarno, Matteo Cesarotto, Luca Salice, Tommaso Bruscagin, Davide Radaelli, Gianfranco Sinagra, Anita Galic Mihic, Maria Assunta Cova, Stefano D’Errico

**Affiliations:** 1 Radiology Department, Azienda Sanitaria Universitaria Giuliano Isontina (ASUGI), University of Trieste, 34127 Trieste, Italy; lorenzo.pagnan@asugi.sanita.fvg.it (L.P.); alessandro.sarno@asugi.sanita.fvg.it (A.S.); matteo.cesarotto@asugi.sanita.fvg.it (M.C.); luca.salice@asugi.sanita.fvg.it (L.S.); m.cova@fmc.units.it (M.A.C.); 2 Forensic Medicine Department, Azienda Sanitaria Universitaria Giuliano Isontina (ASUGI), University of Trieste, 34127 Trieste, Italy; tommaso.bruscagin@studenti.units.it (T.B.); davide.radaelli@units.it (D.R.); 3 Cardiothoracovascular Department, Azienda Sanitaria Universitaria Giuliano Isontina (ASUGI), University of Trieste, 34127 Trieste, Italy; gianfranco.sinagra@asugi.sanita.fvg.it; 4 Institute of Forensic Medicine and Criminalistics, School of Medicine, University of Zagreb, 10000 Zagreb, Croatia; anita.galic@mef.hr

**Keywords:** sudden cardiac death, post-mortem cardiac MR, forensic pathology, autopsy, Friuli Venezia Giulia Registry

## Abstract

**Objectives**: Sudden cardiac death (SCD) is a leading cause of mortality, accounting for approximately 50% of all cardiovascular deaths and 20% of all-natural deaths in Western countries. In individuals over 50 years of age, coronary artery disease (CAD) is responsible for more than 80% of cases, whereas in younger subjects SCD is more frequently associated with non-ischemic myocardial diseases, including hypertrophic cardiomyopathy (HCM), arrhythmogenic cardiomyopathy (ACM), dilated cardiomyopathy (DCM), and myocarditis. Additional causes in young adults include coronary artery anomalies and primary arrhythmic disorders related to channelopathies. This study evaluated the diagnostic performance of post-mortem cardiac magnetic resonance imaging (PM-CMR) in identifying morphological substrates underlying SCD in formalin-fixed explanted hearts, with particular attention to the concordance between PM-CMR findings and autopsy results in cases of sudden coronary death. **Material and Methods**: We retrospectively reviewed 110 PM-CMR examinations from the Regional Register of Sudden Cardiac Death of Friuli-Venezia Giulia, of which 101 were included in the final analysis. **Results**: PM-CMR detected pathological findings in 60 hearts (59%), including acute ischemic lesions in 39 cases and other conditions, such as hypertrophic cardiomyopathy, chronic fibrotic ischemic changes, and adipose metaplasia in 21 cases. A good agreement between PM-CMR and autopsy findings was observed (Cohen’s kappa = 0.8). **Conclusions**: Overall, PM-CMR proved effective in identifying relevant morphological and signal alterations, supporting conventional autopsy. Despite some limitations, particularly in hyperacute ischemic lesions, PM-CMR appears to play a promising role in the diagnostic work-up of SCD and in supporting family screening programs for primary prevention.

## 1. Introduction

Sudden cardiac death is defined as death from cardiovascular or unexplained causes occurring within one hour of symptom onset or within 24 h of last being seen alive in unwitnessed cases. SCD represents the leading cause of sudden death [1], accounting for approximately 50% of cardiovascular deaths and 20% of all-natural deaths in Western countries [2,3]. In individuals over 50 years of age, coronary artery disease is responsible for more than 80% of cases in developed countries. In younger subjects, SCD more often results from non-ischemic myocardial diseases, such as hypertrophic cardiomyopathy, arrhythmogenic cardiomyopathy, dilated cardiomyopathy or myocarditis. Other causes in young adults include coronary artery anomalies and channelopathy-related arrhythmias [4,5,6]. Currently, post-mortem pathological and forensic examination, supported by clinical history, are considered the gold standard for determining the cause of SCD [7]. However, over the past decade, pre-autopsy PM-CMR has emerged as a valuable complementary tool in forensic investigations of cardiovascular-related deaths, as it can detect focal myocardial abnormalities that could be missed at autopsy [8,9]. By identifying these subtle lesions, PM-CMR can significantly improve diagnostic accuracy [10] and has increasingly been adopted as a standard procedure [11,12]. The aim of this study was to evaluate the effectiveness of PM-CMR in identifying morphological substrates responsible for SCD in formalin-fixed explanted hearts, with a specific focus on the concordance between PM-CMR findings and autopsy results in cases of sudden coronary death.

## 2. Materials and Methods

Between May 2021 and December 2025, a total of 110 PM-CMR examinations were performed on consecutive explanted hearts, fixed in 10% buffered formalin, retrieved from the Regional Register of Sudden Cardiac Death in Friuli-Venezia Giulia [13]. Nine specimens were excluded from the analysis, since two hearts were in an advanced state of decomposition and seven were non-diagnostic due to excessive imaging artifacts. Pre-analytical information included: sex, age, risk factors for SCD, non-cardiac comorbidities, family history of sudden death, ischemic heart disease, myocarditis, arrhythmias, or other cardiovascular risk factors, a preliminary report of gross examination from autopsy and post-mortem toxicological analysis on blood and urine samples.

The study population included 101 individuals who died suddenly: 82 males (81%) and 19 females (19%) with a median age of approximately 42 years (range 18–57). Although the Regional Registry is formally designed to include cases of sudden death occurring in individuals aged 0–50 years, a limited number of subjects older than 50 years (up to 60 years of age) were exceptionally included. In these cases, the available clinical history was negative for previously diagnosed cardiac disease, and the circumstances of death raised the possibility of an underlying inherited cardiac condition. The inclusion of these selected cases was based on the preventive and familial screening objectives that constitute one of the fundamental purposes of the Registry. Indeed, the identification of potentially heritable cardiac disorders in victims of sudden death may provide clinically relevant information for the assessment and management of surviving relatives at risk, even when the deceased falls marginally outside the predefined age criteria.

From a medical and family history perspective, most subjects had no risk factors for SCD (60 subjects, 59% of the total population); 6 subjects (6%) had a positive family history for SCD, and 31 subjects (31%) presented cardiovascular risk factors such as hypertension, diabetes mellitus, obesity or epilepsy. Toxicological screenings were performed in 98 subjects: 69 (70%) were negative, while 29 (30%) were positive.

### 2.1. Fixation Protocol

The interval between death and autopsy, with subsequent heart explantation and fixation, varied according to the circumstances of death and, in cases undergoing judicial investigation, according to the administrative and procedural requirements necessary to authorize post-mortem examination. Nevertheless, with the exception of a limited number of cases, the post-mortem interval between death and the initiation of tissue fixation was generally comprised between 3 and 5 days. This variability reflects the routine operational workflow of both forensic and pathological investigations and is representative of real-world conditions encountered in the management of sudden death cases included in the Registry.

All hearts were fixed according to the standardized protocol adopted by the Regional Registry. Following explantation during autopsy, the specimens were thoroughly rinsed with running water to remove residual intravascular blood and minimize fixation-related artifacts. Subsequently, a small opening was created in the ascending aortic wall, through which a supporting thread was passed. This procedure allowed the heart to be suspended within the fixation container, thereby preventing direct contact with the bottom or lateral walls of the vessel and reducing the risk of deformation of the cardiac chambers during the fixation process. The suspended heart was then placed in a dedicated container and immersed in 5 L of 4% buffered formalin using an automated filling system. The specimen was positioned to allow free circulation of the fixative through the cardiac cavities, ensuring complete filling of the chambers and promoting homogeneous tissue fixation throughout the entire organ. No replacement of the fixative solution was performed during the fixation period. All specimens remained continuously immersed and suspended in the same buffered formalin solution for a period ranging from 10 to 15 days prior to post-mortem magnetic resonance imaging (PM-MRI) examination.

### 2.2. Technical Parameters and Examination Protocol

All the examinations were acquired in the Radiology Department of our hospital in Trieste, using either a 1.5 T (Philips Gyroscan Achieva, Philips Medical System) or a 3.0 T superconductive magnet (Ingenia 3.0 T, Philips Medical Systems, Eindhoven, The Netherlands). All MRI examinations were conducted under controlled environmental conditions, with a constant room temperature maintained throughout the study period. The study protocol included the acquisition of a conventional 3D “scout view” localization sequence using a 32-channel surface coil. From a 4 and a 2-chamber b-FFE scan, two series of short-axis views, including both atria and ventricles, were acquired using TSE T1 and T2 weighted Dixon sequences, with and without fat suppression. A total of 3 short-axis slices of mapping (MOLLI T1; BB-GraSE T2 and ME-TFE T2*) were acquired in the basal, middle and apical heart segments. A 3D balanced-FFE (SSFP T2/T1) was finally acquired to visualize the coronary arteries. Total examination time lasted approximately 30 min. (Table 1).

For each T1, T2 and T2* mapping image, endocardial and epicardial contours were traced manually. Post-processing analysis for T1, T2 and T2* mapping was performed using IntelliSpace Portal 9.0 (Philips, Amsterdam, The Netherlands) software. Native T1, T2 and T2* maps were then automatically calculated on basal, mid and apical LV short-axis slices. The myocardium was automatically segmented into American Heart Association (AHA) segments. Images from explanted hearts were then analyzed by two readers experienced in cardiac radiology to evaluate the presence or absence of signal alterations and their localization.

Myocardial signal intensity alterations detected on PM-CMR were correlated with myocardial lesions at macroscopic autopsy, histological examination or coronary findings. Cohen’s kappa coefficient was used to measure inter-rater reliability between radiological and autopsy findings.

Acute ischemic lesions were defined as those exhibiting a coronary distribution, subendocardial extension, crescent-shaped morphology, and hyperintense, heterogeneous signal on T1 and/or T2-weighted images. Chronic fibrotic lesions were defined as those with hypointense homogeneous signal on T1-weighted and iso- to slightly hyperintense signal on T2-weighted images. Metaplastic and lipomatous areas were defined as hyperintense on T1 and T2-weighted images and hypointense on fat-suppressed T1 sequences.

## 3. Results

PM-CMR revealed pathological findings in 60 explanted hearts (59% of the total population), including ischemic lesions in 39 cases, and other non-ischemic findings—such as wall hypertrophy, fibrotic lesions and adipose metaplasia—in 21 cases. No anomalies in the origin of the coronary arteries were detected. Autopsy attributed a coronary-related etiology to SCD in 45 cases (45%) and a non-coronary etiology in 56 cases (55%).

**Figure 1:**
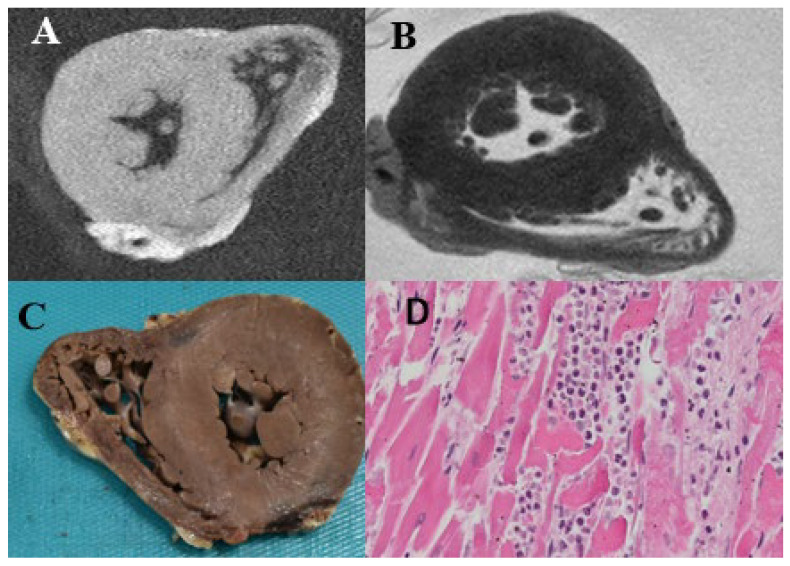
T2- (**A**) and T1-weighted (**B**) short-axis cardiac images showing preserved signal intensity despite proximal occlusion of the left anterior descending (LAD) coronary artery. Gross pathological (**C**) and histological (**D**) analyses revealed an ischemic area in the posterior region with inflammatory infiltrate; findings consistent with hyperacute ischemic injury undetectable in T1- and T2-weighted images acquired (20×).

A total of 62 explanted hearts (61%) were negative for ischemic lesions on PM-CMR, and 55 cases (54%) were confirmed as negative at autopsy, while seven cases (7%) were attributed to coronary-related death and were therefore classified as false negatives. 39 explanted hearts (39%) were positive for ischemic injuries on PM-CMR and among them, 37 cases (37%) were confirmed at autopsy, whereas two cases (2%) were not confirmed and were classified as false positives. In one of the two discordant cases, PM-CMR failed to identify a hyperacute myocardial ischemic injury. As shown in Figure 1, T1- and T2-weighted post-mortem cardiac MR images demonstrated preserved myocardial signal intensity despite proximal occlusion of the left anterior descending (LAD) coronary artery. Gross pathological and histopathological examinations subsequently revealed an ischemic myocardial area with early inflammatory infiltrate, consistent with hyperacute ischemic injury. This case underscores the limited sensitivity of conventional PM-CMR sequences in detecting very early ischemic changes, which may only become evident at autopsy and histological examination.

All five cases of hypertrophic cardiomyopathy were correctly identified and subsequently confirmed at histopathological examination, whereas one case of myocarditis was not detected by PM-CMR. The remaining 15 cases were characterized by adipose or fibrotic replacement/metaplasia, which were accurately recognized on post-mortem imaging and corroborated by conventional autopsy and histological findings.

An analysis of concordance between PM-CMR and autopsy in identifying coronary artery disease as the cause of sudden death was performed. The calculated Cohen’s kappa coefficient was 0.8, indicating good agreement between radiological and pathological findings. In our cohort, no appreciable differences in diagnostic specificity or overall diagnostic yield were detected between PM-CMR examinations performed on 1.5-T and 3-T scanners. The field strength did not significantly affect the identification of cardiac structural abnormalities or the subsequent diagnostic interpretation. Therefore, both imaging platforms appear equally suitable for post-mortem cardiac assessment when integrated into the forensic investigation of sudden cardiac death.

## 4. Discussion

With the Regional Law No. 26 [13,14], enacted on 30 December 2020, the Friuli-Venezia Giulia Region established the Regional Register of Sudden Cardiac Death to support a systematic investigation of deaths occurring suddenly or unexpectedly in individuals under 50 years of age, for whom the cause of death cannot be determined with certainty. With this diagnostic work-up PM-CMR on the explanted heart was introduced prior to autopsy and pathological examination.

To understand this peculiar branch of radiological imaging, it is essential to consider the effects of physiological post-mortem processes on imaging. Metabolic shut-down at death leads to a progressive decrease in body temperature, which significantly affects PM-CMR image contrast, as both T1 and T2 relaxation times are temperature-dependent parameters [15]. According to Ruder et al., the contrast between fat and muscle tissue on T2-weighted images is directly proportional to body temperature, whereas the contrast between adipose tissue and fluids is inversely proportional. Below 20 °C, the contrast between adipose and muscle tissue is lost on T2-weighted images. Similarly, low body temperature also results in reduced contrast on T1-weighted images [16].

Putrefaction represents another factor influencing post-mortem imaging. Gas formation, resulting from the catabolic activity of anaerobic bacteria originating from the intestinal flora, occurs as these microorganisms reach the heart after death. Initially, small gas bubbles develop within the ventricular wall, first appearing in the endocardium and eventually spreading through the full thickness of both ventricles, causing pronounced low-signal artifacts [17].

**Figure 2:**
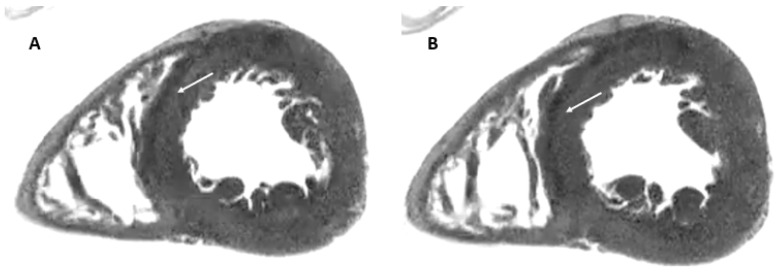
T2-weighted PM-CMR images of a formalin-fixed heart showing hypointense striae (arrows), consistent with hypostasis (**A**,**B**).

Rigor mortis also affects post-mortem imaging. Depletion of myocardial ATP stores causes the heart to remain in systole, leading to increased intramyocardial pressure from the subepicardial to the subendocardial layers. This pressure gradient promotes interstitial fluid shifts toward the lower-pressure subepicardial layers, where internal livores may appear as hypointense areas on T2-weighted images (Figure 2). This process results in altered signal intensity from subepicardial to subendocardial regions when rigor mortis is fully expressed (24–48 h) [18].

Additional factors influencing post-mortem imaging include intravascular coronary clot formation following cessation of blood circulation [19].

PM-CMR can be performed either on the whole cadaver or on a formalin-fixed explanted heart. Most published PM-CMR studies have been conducted on cadavers prior to autopsy, whereas only a limited number have been performed on formalin-fixed explanted hearts. Formalin further alters the magnetic properties of myocardial tissue, primarily due to dehydration, which modifies native myocardial relaxation times and hampers the detection of the small amount of edema typically present in hyperacute myocardial infarction or myocarditis.

In our experience, the native T1 value of normal myocardium decreased to approximately 440 ms, compared with 1050 ms observed in vivo at 1.5 T and 1260 ms at 3 T, whereas T2 values remained around 50 ms both at 1.5 T and at 3 T, similar to those observed in vivo. These alterations may partly explain the limited number of published PM-CMR studies performed on formalin-fixed specimens.

Nevertheless, examining an explanted heart offers substantial logistical advantages over performing MR on a whole cadaver, as it avoids body transportation and minimizes interference with routine diagnostic activities. Despite these technical challenges, preliminary studies have demonstrated a strong correlation between autopsy and PM-CMR findings in cases of myocardial infarction [20].

**Figure 3:**
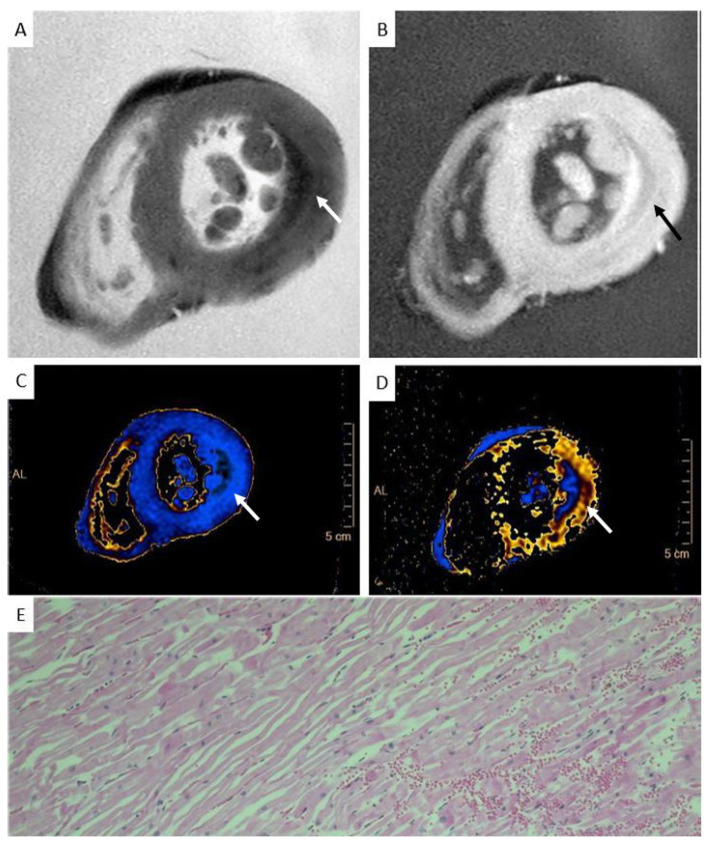
Formalin-fixed heart from a 49-year-old woman who died of cardiac arrest due to myocardial infarction. PM-CMR shows a hypointense subendocardial crescent-shaped lesion in the posterolateral wall of the left ventricle on fat-suppressed T2-weighted image (**A**), with a corresponding hypointense signal surrounded by a hyperintense rim on fat-suppressed T1-weighted images (**B**). T2 and T2* mapping demonstrate reduced values within the ischemic core, consistent with haemorrhagic infiltration (**C**,**D**). Histological examination confirms the presence of widespread interstitial haemorrhagic components (10×) (**E**).

**Figure 4:**
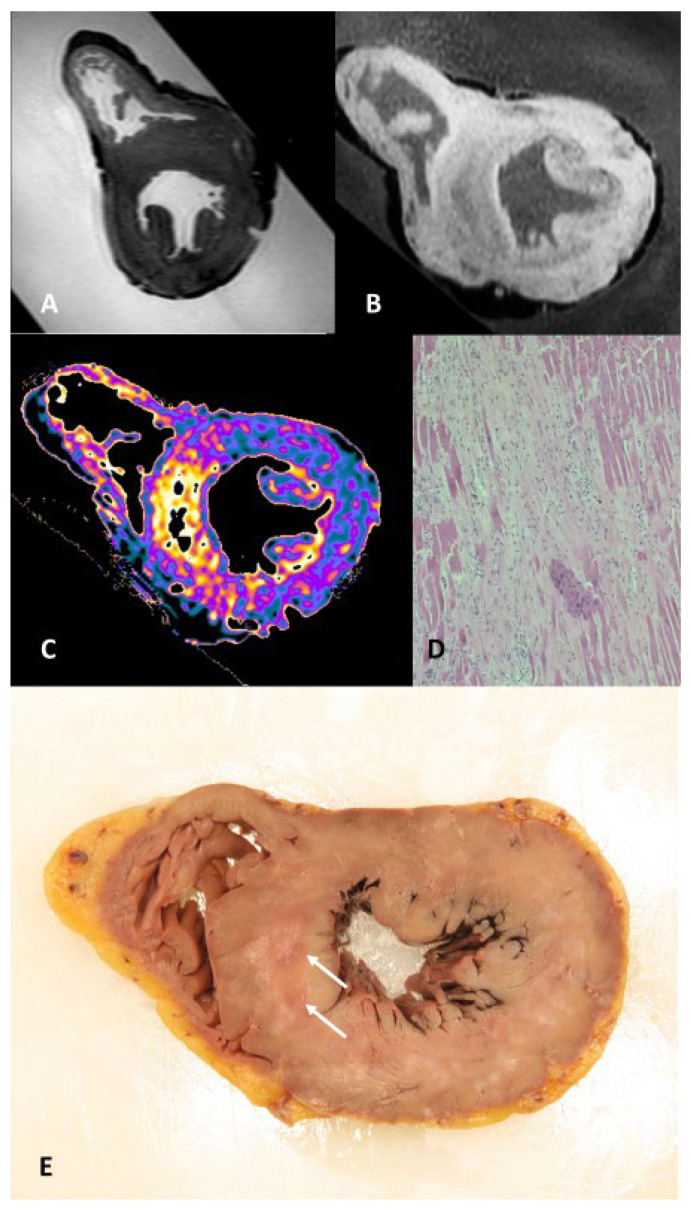
Formalin-fixed heart from a 50-year-old man who died during physical activity. PM-CMR shows an extensive crescent-shaped intramural hypointense area within the mid interventricular septum on fat-suppressed Dixon T1-weighted (**A**) and iso- to slightly hyperintense signal on T2-weighted images (**B**). T1 mapping (**C**) demonstrates increased values consistent with fibrotic tissue, subsequently confirmed by histological examination (10×) (**D**), the myocardial fibrosis is also macroscopically visible ((**E**), white arrows).

**Figure 5:**
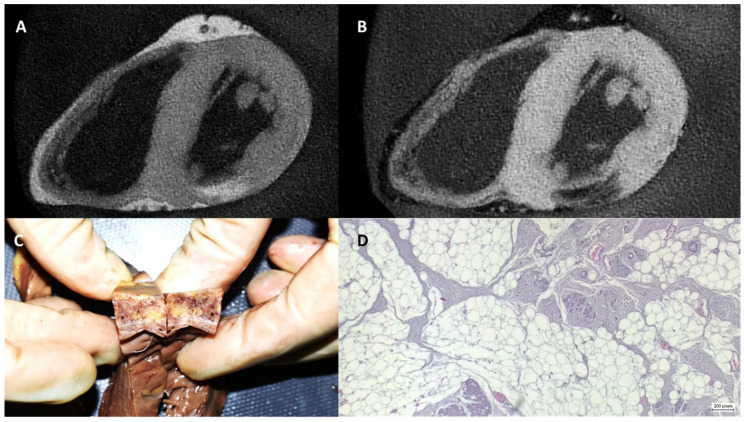
Sudden cardiac death of a 45-year-old man with a family history of not investigated sudden death (both parents), found dead at home. PM-CMR of explanted and fixed in formalin heart showed extensive fatty infiltration of the infero-medio-basal wall of the left ventricle on T1-weighted Dixon with and without fat suppression images (**A**,**B**), confirmed by autopsy and histological findings (10×), demonstrating adipose metaplasia and severe coronary atherosclerosis (**C**,**D**).

Hyperacute myocardial infarction is generally not detectable at gross examination when death occurs within the first 12 h of the ischemic event. However, when survival ranges between 2 and 6 h, histopathological changes such as hypereosinophilia, loss of striations, and a wavy appearance of myocardial fibers may be observed [10]. In our cohort, no hyperacute ischemic lesions were detected. Acute ischemic lesions appeared as heterogeneous hyperintense signal abnormalities on T1- and/or T2-weighted images, with coronary distribution, subendocardial extension, and crescentic morphology (Figure 3). Chronic fibrotic lesions appeared homogeneously hypointense on T1-weighted and iso- to slightly hyperintense signal on T2-weighted images (Figure 4), whereas areas of adipose metaplasia showed hyperintensity on T1- and T2-weighted images and signal loss on fat-suppressed Dixon acquisitions (Figure 5).

**Figure 6:**
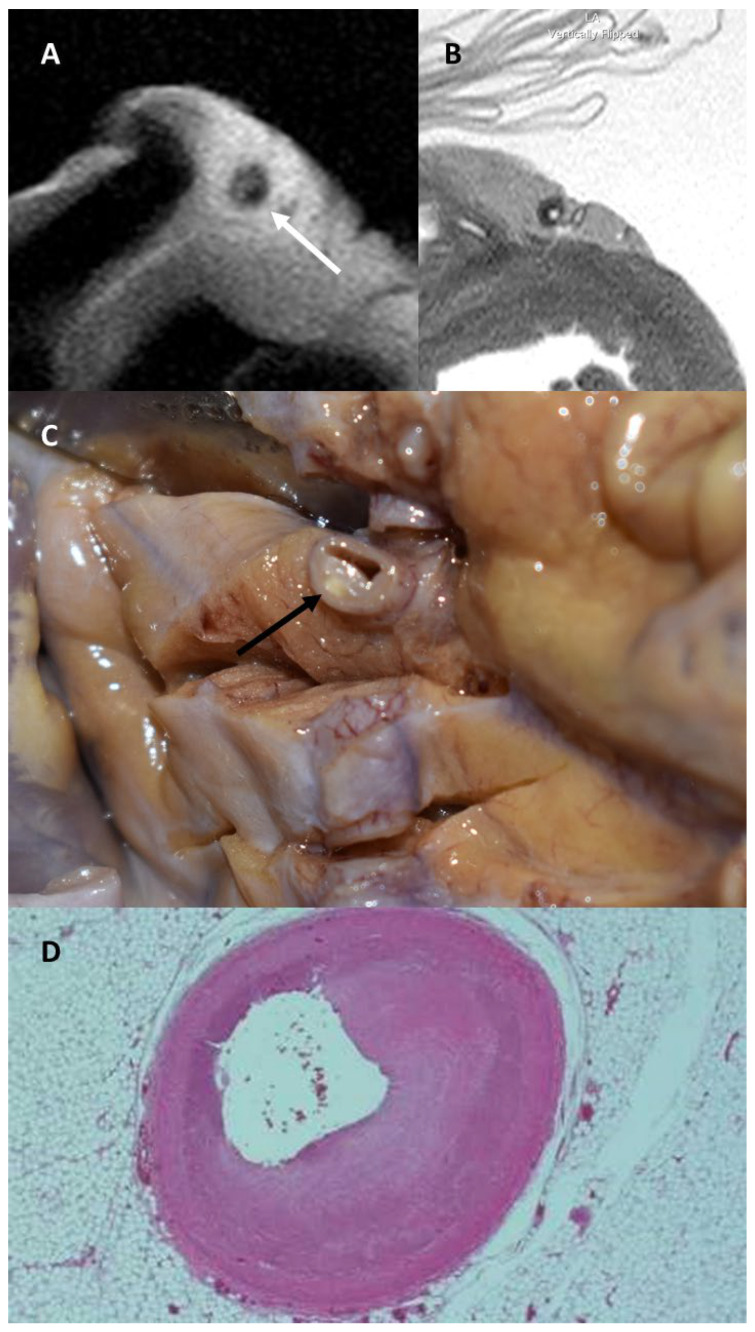
Sudden cardiac death in a 48-year-old man. PM-CMR of a formalin-fixed explanted heart showing an atheromatous plaque (arrows) of the left main coronary artery on T1-weighted (**A**) and T2-weighted (**B**) images. Section of formalin-fixed explanted heart showing the same atheromatous plaque of the left main coronary artery (**C**). Histological sections of an atheromatous plaque from the same explanted heart are shown in (**D**) (4×).

Although assessment of coronary artery disease using non-contrast PM-CMR remains challenging, the three-dimensional steady state free precession (3D-SSFP) sequence enabled improved anatomical evaluation of the coronary arteries, while T2-weighted sequences facilitated visualization of parietal atheromatosis in proximal segments (Figure 6).

The primary goal of our study was to evaluate the effectiveness of PM-CMR in determining the cause of SCD, with coronary artery disease representing the most expected etiology. PM-CMR demonstrated high diagnostic accuracy, comparable to autopsy, especially in the identification of ischemic myocardial lesions. A good concordance was also observed between radiological and histopathological findings, as well as in the exclusion of pathological substrates. In our series, seven cases showed negative PM-CMR findings despite positive coronary findings at autopsy. This discrepancy was mainly related to hyperacute ischemic lesions, in which histopathological alterations were present but not yet detectable on MR, likely due to formalin-induced dehydration. Conversely, only two false-positive cases of ischemic death were observed, both probably related to incomplete formalin fixation.

Accurate evaluation of coronary arteries remains crucial in PM-CMR. Although thrombus detection on T2-weighted sequences is highly specific, its sensitivity is low [21]. In our cohort, assessment of coronary origin and course using 3D-SSFP sequence proved most effective to evaluate vessel patency, whereas T2-weighted imaging was more useful for detecting pathological wall alterations of proximal coronary arteries.

**Figure 7:**
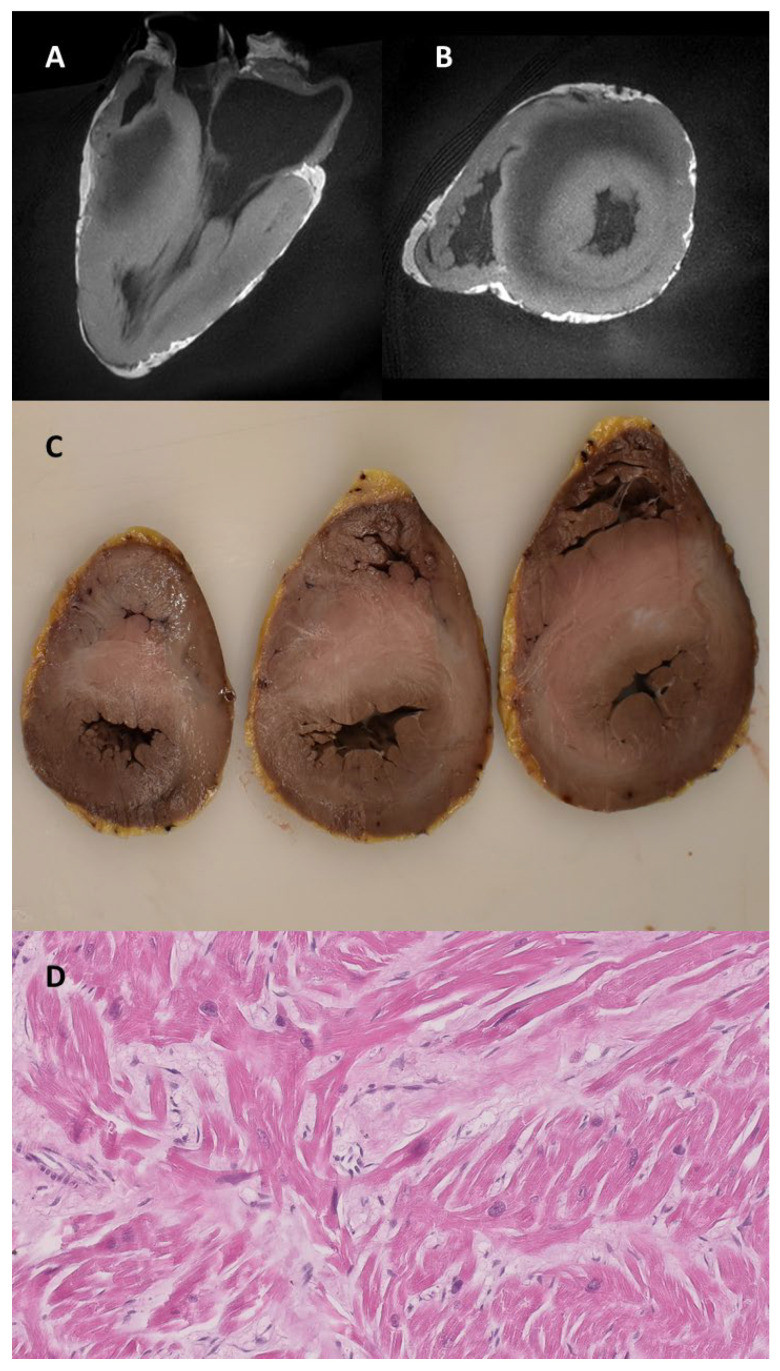
Sudden cardiac death in a 23-year-old man found lifeless at home. PM-CMR of a formalin-fixed heart with hypertrophic cardiomyopathy, marked septal hypertrophy on four-chamber long-axis (**A**) and short-axis T1 (**B**). After dissection of the heart, hypertrophy of left ventricle walls and interventricular septum were confirmed (**C**). Myocardium disarray, hypertophic myocites and focal interstitial fibrosis completed histological panel of findings (20×) (**D**).

Only a few studies have reported the use of post-mortem MR in the evaluation of cardiomyopathies, as autopsy and histology remain the diagnostic gold standards. Nevertheless, PM-CMR may play a supportive role in diagnosing HCM and ACM, especially in identifying asymmetric myocardial thickening, ventricular fatty infiltration, or apical thinning of the right ventricle (Figure 7) [22]. In our experience, all cases of HCM were correctly identified, whereas a single case of myocarditis-related death was not detected by PM-CMR, likely due to dehydration effects of formalin fixation.

Several limitations of this study should be acknowledged. Limited knowledge remains regarding the effects of formalin fixation on myocardial T1 and T2 relaxation times and the influence of temperature on tissue characteristics. Rigor mortis may further complicate the differential diagnosis with HCM. Finally, PM-CMR shows limited accuracy in evaluating distal coronary vessels and in distinguishing true coronary occlusion from post-mortem endoluminal clot formation. In this context, post-mortem CT angiography should be considered a complementary technique [23,24,25].

To enhance the clinical applicability of this approach, we developed an operational algorithm for the integration of post-mortem cardiovascular magnetic resonance (PM-CMR) into the diagnostic workflow of sudden cardiac death.

**Figure 8:**
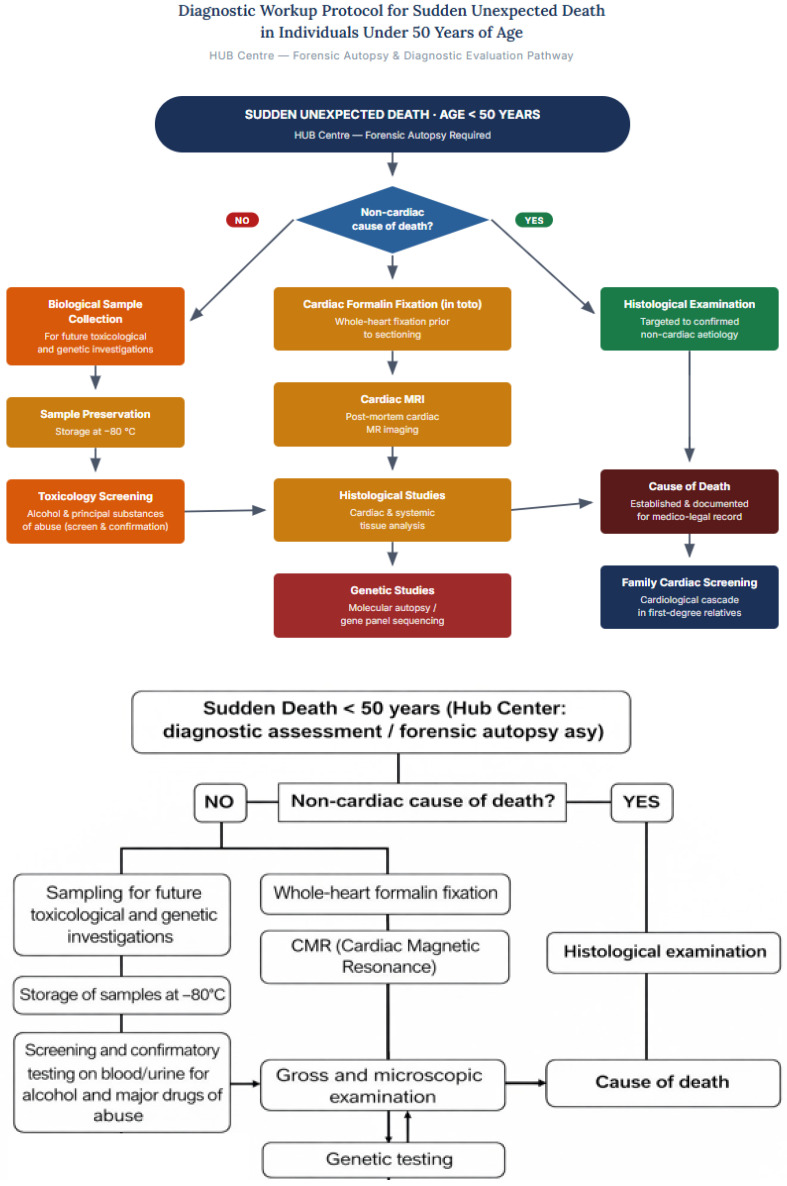
Diagnostic algorithm for the investigation of sudden death in individuals aged <50 years potentially eligible for inclusion in the Friuli Venezia Giulia Regional Registry of Sudden Cardiac Death in the Young. MRI, magnetic resonance imaging.

In our experience, PM-CMR is routinely employed in selected cases of suspected sudden cardiac death as part of an integrated strategy alongside conventional autopsy. PM-CMR provides substantial added value during the preliminary screening and diagnostic orientation phase, enabling the identification of cardiac structural abnormalities that are otherwise difficult to detect or not readily localizable. This contribution is particularly useful in guiding targeted histological sampling, thereby increasing the diagnostic accuracy of the autopsy and optimizing subsequent morphological assessment. The proposed algorithm (Figure 8) [13] therefore reflects an integrated model in which PM-CMR serves as an intermediate step between clinical suspicion and histopathological confirmation, improving case stratification and overall diagnostic yield, particularly in settings where access to full autopsy may be limited or delayed. There is no doubt that the systematic integration of advanced imaging techniques into routine autopsy practice is associated with organizational and economic challenges that are not always easy to overcome. Nevertheless, these challenges should encourage forensic professionals and healthcare stakeholders to engage in a critical reflection aimed at progressively implementing such approaches within modern post-mortem investigations.

The establishment of pathology-specific registries, such as the Friuli Venezia Giulia Regional Registry of Sudden Cardiac Death, should be regarded as an exemplary organizational model capable of fostering collaboration between healthcare institutions, academic centers, and local authorities. Such initiatives justify and support public investment in activities directed toward scientific research, disease prevention, and the protection of public health and citizen safety.

## 5. Conclusions

Autopsy and pathological examination of the explanted heart remain the gold standard for determining the etiology of SCD. However, PM-CMR in formalin-fixed explanted hearts represents a practical and promising technique in this diagnostic work-up, owing to its ability to detect myocardial signal and morphological alterations able to guide pathological examination. Nevertheless, formalin-induced tissue dehydration can impair image interpretation, especially in cases of hyperacute ischemia and myocarditis. Assessment of coronary artery patency also remains critical in PM-CMR and, as it is essential in correlating imaging findings with myocardial ischemia, complementary post-mortem CT angiography should be considered.

## Figures and Tables

**Table 1:** PM-CMR protocol: sequences and MR parameters.

Short Axis (Acquisition Time 20 m)	b-FFE	T1 (Dixon)	T2 (Dixon)	MOLLI (T1 Mapping)	m-GRASE (T2 Mapping)	T2* Map (T2* Mapping)
**TR (ms)**	5.5	550	3420	2.8	1000	14.4
**TE (ms)**	1.47	16	100	1.31	14	6.6
**FA (°)**	90	90	90	35	90	20
**Slice thickness (mm)**	5	3	3	5	5	5
**FOV**	200 × 200	160 × 160	160 × 160	170 × 170	170 × 170	170 × 170

## Data Availability

All data related to the Friuli Venezia Giulia Regional Registry of Sudden Cardiac Deaths are stored in a database to which access is restricted to the Registry’s stakeholders and protected by a password. The data can be exported to an Excel file upon formal request by the publisher to the corresponding author.

## Abbreviations

The following abbreviations are used in this manuscript:

SCD	Sudden Cardiac Death
CAD	Coronary Artery Disease
HCM	Hypertrophic Cardiomyopathy
ACM	Arrhythmogenic Cardiomyopathy
DCM	Dilated Cardiomyopathy
PM-CMR	Post-Mortem Cardiac Magnetic Resonance Imaging
AHA	American Heart Association
MR	Magnetic Resonance Imaging
